# Complete genome sequence of bacteriophage LMC infecting *Listeria monocytogenes* from fowl droppings

**DOI:** 10.1128/mra.00333-25

**Published:** 2025-05-22

**Authors:** Jiyoon Chung, Yoonjee Chang

**Affiliations:** 1 Department of Food and Nutrition, College of Science and Technology, Kookmin University, Seoul, South Korea; Queens College, Queens, New York, USA

**Keywords:** *Listeria* phage, antibacterial activity, genomic characterization, biocontrol agent, *Listeria monocytogenes*

## Abstract

*Listeria monocytogenes*-targeting phage (LMC) was isolated from fowl droppings, and its complete genome was analyzed. LMC consists of 42,151 bp with a GC content of 36.58% and contains 59 open reading frames. It has an icosahedral capsid and a long tail. It shows a high antibacterial ability against *L. monocytogenes* ATCC 19115.

## ANNOUNCEMENT

*Listeria* is a Gram-positive genus that causes food poisoning (1). *Listeria monocytogenes* can adapt effectively to low-temperature, high-salinity environments and has high biofilm-forming ability (2). *L. monocytogenes* can be preserved in the global food chain with these characteristics. Consequently, *Listeria* contamination continues to be a problem in food production (3). To control this bacterium, substances such as antibiotics can be used, but it can lead to the development of antibiotic-resistant bacteria (4). Therefore, current studies focus on bacteriophages that can effectively control this bacterium without causing any antibiotic resistance (5).

**Fig 1:**
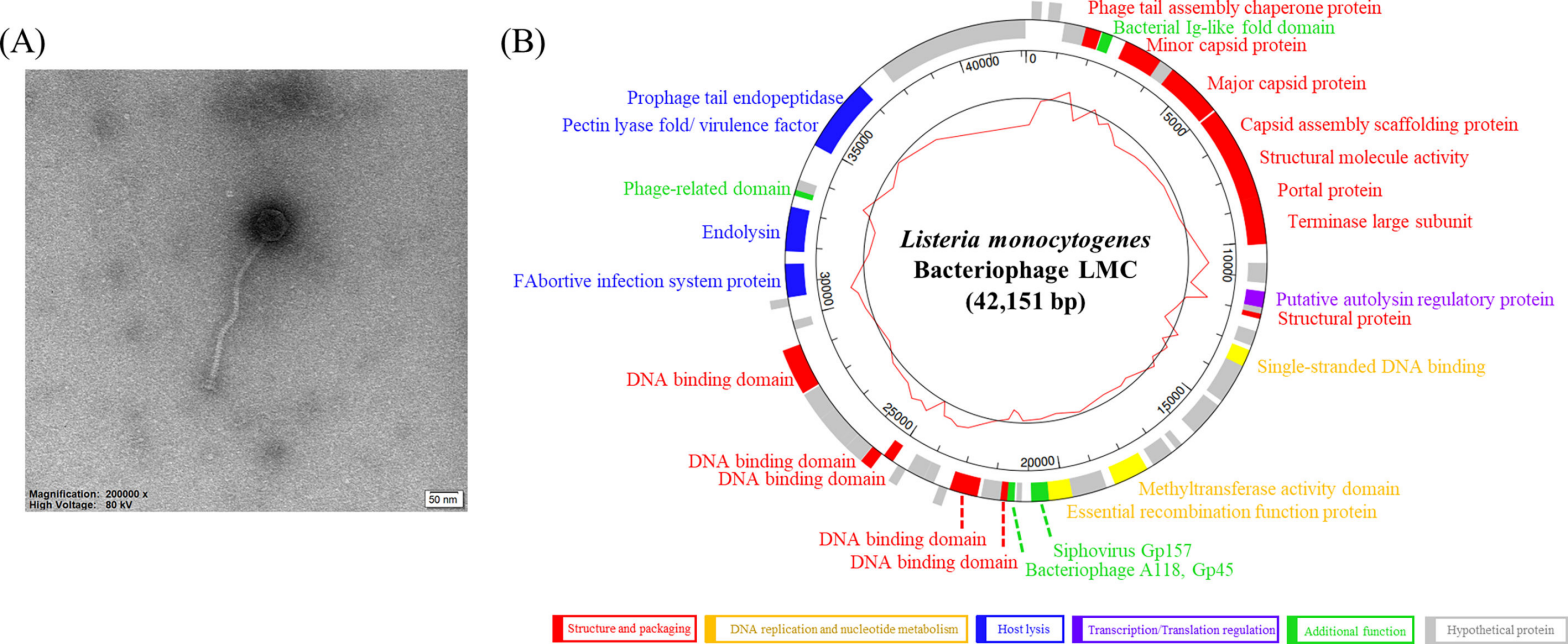
(**A**) TEM image of phage LMC (measured from six independent replicates). The scale bar means 50 nm. (**B**) The genomic map of phage LMC. The outer circle shows the ORFs, and specific colors indicate the group with specific functions. The inner circle indicates the GC content. Each ORF is classified into six types and analyzed: (ⅰ) structure and packaging, (ⅱ) DNA replication and nucleotide metabolism, (ⅲ) host lysis, (ⅳ) transcription/translation regulation, (ⅴ) additional function, and (ⅵ) hypothetical protein.

This study aimed to isolate a new bacteriophage (phage) targeting *Listeria* and to analyze its characteristics. Phages can be isolated from environments rich in bacteria (6). Considering that *L. monocytogenes* is frequently associated with poultry, fecal samples from poultry were selected for phage isolation. LMC was isolated from poultry droppings using its host strain, *L. monocytogenes* ATCC 19115, in Dongtan, South Korea (37.2056°N, 127.0789°E). The host was cultured in brain heart infusion medium under aerobic conditions at 37°C. After purification, LMC produced a single, well-defined plaque on the overlaid host lawn, confirming its identity. Morphology was visualized by transmission electron microscopy (TEM) following negative staining with 2% uranyl acetate. Phage dimensions were measured from six independent replicates. Total genomic DNA was extracted using the phenol–chloroform method. Sequencing was performed on an Illumina HiSeq platform using 151 base pair (bp) paired-end reads, following library preparation with the TruSeq Nano DNA Library Prep Kit (Illumina, USA). Quality control was performed using FastQC version 0.11.5 (7), and trimming was done with Trimmomatic version 0.36 (8) under default settings. The average sequencing depth was 139×. The reads were assembled *de novo* using SPAdes version 3.15.5. Open reading frames (ORFs) were analyzed using GeneMarks, FgenesV software, and Rast, and annotated via InterProScan (https://www.ebi.ac.uk/interpro) and BLASTP (https://blast.ncbi.nlm.nih.gov/Blast.cgi). The genome map was generated using GeneScene version 0.99.8.0. The host range was confirmed via a spot assay. Phage lysate was dropped onto the lawn containing the host strain, and the extent of host lysis was measured. TEM revealed that LMC has an icosahedral capsid and a long tail, indicating it belongs to the *Caudoviricetes* class (Fig. 1A). The capsid size of LMC is approximately 57.35 ± 1.98 nm, and the tail width and length are 12.60 ± 1.00 nm and 288.98 ± 0.35 nm, respectively. LMC consists of a linear double-stranded DNA with 42,151 bp. It contains 36.58% GC and 59 ORFs (Fig. 1B). Specifically, 15 ORFs contribute to phage structure and packaging. Next, 3 ORFs are related to DNA replication, and 5 ORFs to host lysis. One ORF is assigned to transcriptional regulation. A total of 31 ORFs are implicated in hypothetical proteins, whereas 4 ORFs showed additional functions. The host lytic ability can be verified by the host range analysis (Table 1). LMC evidently and clearly killed *L. monocytogenes* ATCC 19115 with significant activity. Thus, the results of this study offer the first full sequencing and characteristics of a phage known as LMC, which can serve as a basis for further phage research.

**TABLE 1:** The host range of phage LMC against *Listeria* strain^*b*^

No.	Strains			Efficiency of plating (EOP)^*a*^
1	*Listeria*	*monocytogenes*	ATCC 19115	++
2			20800818	+
3			ATCC 19111	−
4			LM-1 12	−
5			LM-1 16	−
6			KCCM 40307	−
7			20800013	−
8			20800014	−
9			20800015	−
10			20800016	−
11			20800018	−
12			20800019	−
13			20800020	−
14			20800021	−
15			20800022	−
16			20800023	−
17			20800024	−
18			DH-1	−
19			DH-2	−
20			DH-3	−
21			DH-4	−
22		*innocua*	ATCC 33090	+
23			KCTC 3586	+
24			ATCC 51742	−

^*a*^ ++, EOP more than 1; +, EOP less than 1; and –, no inhibition.

^*b*^ Higher EOP indicates stronger lytic activity.

## Data Availability

The entire genome sequence and genetic analysis data of *Listeria monocytogenes* target phage LMC are submitted to GenBank (accession number: PQ869677). The associated BioProject, BioSample, and SRA accession numbers are PRJNA1236025, SAMN47378814, and SRR32695915, respectively.
